# Social odours covary with bacterial community in the anal secretions of wild meerkats

**DOI:** 10.1038/s41598-017-03356-x

**Published:** 2017-06-12

**Authors:** Sarah Leclaire, Staffan Jacob, Lydia K. Greene, George R. Dubay, Christine M. Drea

**Affiliations:** 1 Centre d’Ecologie Fonctionnelle et Evolutive, UMR 5175, CNRS, 1919 route de Mende, 34293 Montpellier, France; 2 Department of Zoology, University of Cambridge, Downing Street, Cambridge, CB2 3EJ UK; 3 Laboratoire Evolution & Diversité Biologique, UMR 5174 (CNRS, Université Paul Sabatier, ENFA), 118 rte de Narbonne, 31062 Toulouse, France; 4 Kalahari Research Trust, Kuruman River Reserve, 8467 Van Zylsrus, Northern Cape South Africa; 5 Department of Evolutionary Anthropology, Duke University, Durham, NC 27708-0383 USA; 6 Department of Chemistry, Duke University, Durham, NC 27708-0383 USA; 7 Department of Biology, Duke University, Durham, NC 27708-0383 USA; 8 Université Catholique de Louvain, Earth and Life Institute, Biodiversity Research Centre, Croix du Sud 4, L7-07-04, 1348 Louvain-la-Neuve, Belgium

## Abstract

The fermentation hypothesis for animal signalling posits that bacteria dwelling in an animal’s scent glands metabolize the glands’ primary products into odorous compounds used by the host to communicate with conspecifics. There is, however, little evidence of the predicted covariation between an animal’s olfactory cues and its glandular bacterial communities. Using gas chromatography-mass spectrometry, we first identified the volatile compounds present in ‘pure’ versus ‘mixed’ anal-gland secretions (‘paste’) of adult meerkats (*Suricata suricatta*) living in the wild. Low-molecular-weight chemicals that likely derive from bacterial metabolism were more prominent in mixed than pure secretions. Focusing thereafter on mixed secretions, we showed that chemical composition varied by sex and was more similar between members of the same group than between members of different groups. Subsequently, using next-generation sequencing, we identified the bacterial assemblages present in meerkat paste and documented relationships between these assemblages and the host’s sex, social status and group membership. Lastly, we found significant covariation between the volatile compounds and bacterial assemblages in meerkat paste, particularly in males. Together, these results are consistent with a role for bacteria in the production of sex- and group-specific scents, and with the evolution of mutualism between meerkats and their glandular microbiota.

## Introduction

Bacteria are ubiquitous and can colonize all habitats, including those occurring within animal bodies^1, 2^. Animals live in association with a suite of microorganisms (called the microbiota) that can affect host life-history traits^3^ and behaviour^4–6^. For instance, bacteria can influence host social behaviour by directly influencing the nervous system^7^ or, more indirectly, by affecting chemical cues that animals use to communicate^8^. Indeed, the fermentation hypothesis for animal olfactory signalling has long posited that bacteria metabolize glandular secretions and produce volatile, organic compounds, such as hydrocarbons, fatty acids, wax esters, and sulfur compounds^9–11^, that are used in communication by the host^5, 12, 13^. Despite mounting evidence in support of the fermentation hypothesis, logistical challenges have hindered examining the covariation between bacterial communities inhabiting the scent-producing organs and the chemical diversity of odorants expressed by wild animals.

Evidence in support of the fermentation hypothesis has derived principally from studies that link bacterial action to specific, olfactory-mediated host behaviour or to the production of certain odorants. For instance, researchers have shown that trimethylamine, an odorant that plays a key role in mouse (*Mus musculus*) reproduction, requires commensal bacteria for its production^14^. Likewise, the characteristic odorants of elephant (*Loxodonta africana*) musth have been shown to derive from bacterial metabolisation of fatty acids^15^. Researchers have also inhibited odorant production in Indian mongooses (*Herpestes auropunctatus*) and European hoopoes (*Upupa epops*) by treating the animals’ scent glands with antibiotics^12, 16^. With the advent of new genetic tools, researchers are increasingly able to identify bacterial assemblages in microhabitats. So far, however, in only one study have researchers used deep sequencing of bacterial communities to test for covariation between microbiota and the volatiles associated with scent glands^17^. Here, we likewise test for such covariation in the meerkat (*Suricata suricatta*), a social carnivoran that relies on both intra- and inter-group olfactory communication.

**Figure 1:**
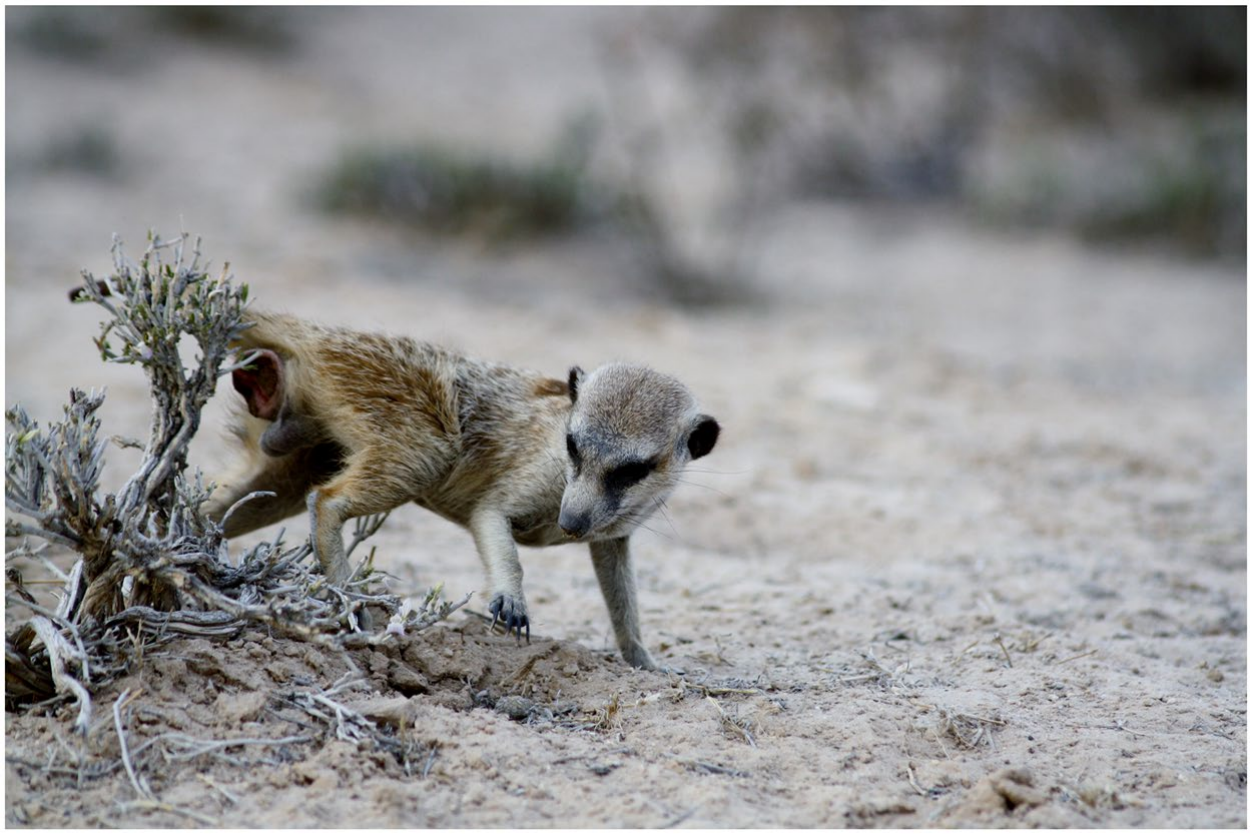
Photograph of a male meerkat everting his anal pouch during scent marking. (Photo courtesy of Lydia K. Greene).

The meerkat is a cooperatively breeding mongoose that uses scent to delineate territories^18^ and communicate social information^19–21^. Animals of both sexes possess anal scent glands that open, via pores, into a large, anal pouch^22^, that is everted during scent marking and rubbed against various substrates (Fig. 1). A liquid secretion (or ‘paste’) can be expressed from these pores (i.e., by squeezing the gland). Moreover, paste accumulates in the pouch, where it can become mixed with faecal material and environmental contaminants (e.g. sand) that adhere to the inside of the pouch during scent marking. In a prior study using a DNA fingerprint method, we confirmed that bacterial communities are present both within the ‘pure’ secretions from the scent glands and within the ‘mixed’ secretions contained in the pouch^23^. Although that approach did not allow for the identification of bacterial phylotypes, we could show that the bacterial communities present within the pouch mixtures varied with host characteristics, such as sex, social status and group membership^23^. To lend further support to the fermentation hypothesis for animal signalling, we now couple deep sequencing techniques with chemical analyses of those secretions, to more directly link host-bacteria relationships to chemical signals.

We first use gas chromatography-mass spectrometry (GCMS) to test for volatile chemical differences between the pure glandular secretions and the mixtures contained within the anal pouch of adult meerkats. Given that bacteria are present in both pure and mixed secretions^23^, we expect both to be populated by bacteria whose taxonomic relatives are well-known odour producers; nevertheless, one might expect an increased contribution from fermenting bacteria in the mixtures. Specifically, we predict a greater representation of high-molecular-weight compounds (that might be endogenously produced in the glands) in pure secretions versus a greater representation of low-molecular-weight (LMW) compounds (that are characteristic of bacterial fermentation)^24^ in the mixtures. Second, because these mixtures are more likely than pure secretions to resemble the actual scent marks that are deposited in the environment, we next relate the volatile chemical profiles of the mixtures to various meerkat attributes, including sex, social status and group membership. Third, using deep sequencing, we identify the bacterial assemblages present in the mixtures and also relate them to the same set of host variables. Lastly, we combine both sets of analyses to test for covariation between the chemical compounds and bacterial assemblages present in mixtures. As in hyaenas^17^ and consistent with the fermentation hypothesis, we expect the bacterial assemblages in meerkat anal-pouch secretions to vary systematically with meerkat social odours.

## Results

### Chemical comparison of secretions derived from the anal gland versus the anal pouch

We mainly detected alcohols, aldehydes, alcanes, carboxylic acids, esterified fatty acids and sterols in the anal-gland secretions of adult meerkats (Table 1). When comparing pure glandular secretions to mixtures obtained from the anal pouch of subordinate meerkats only, we detected a total of 222 different chemical compounds in the 31 samples of pure secretions and, similarly, a total of 218 compounds in the 24 samples of mixed secretions. The richness per sample (i.e., for individual meerkats) was similar in pure and mixed secretions (t_1,52_ = −0.06, P = 0.95; mean richness per individual in subordinate meerkats: 73.8 ± 3.0 compounds in pure secretions and 73.5 ± 3.0 compounds in the mixtures).

**Table 1:** Mean percentage ± SE of the chemical compounds putatively identified in the pure and mixed anal gland secretions of subordinate meerkats.

Fatty acids	Mean percentage ± SE		Mann-Whitney test*
	Pure glandular secretions	Anal-pouch mixtures	
Octanoic acid	y	y	
Nonanoic acid	y	y	
Dodecanoic acid	0.04 ± 0.01	0.19 ± 0.06	W = 270, P = 0.059
Tetradecanoic acid	0.18 ± 0.03	0.13 ± 0.05	W = 524, P = 0.007
Pentadecanoic acid	0.21 ± 0.03	0.09 ± 0.04	W = 558, P = 0.001
9-Hexadecenoic acid	2.11 ± 0.33	0.03 ± 0.03	W = 739, P < 0.0001
Hexadecanoic acid	21.55 ± 1.65	4.41 ± 1.08	W = 705, P < 0.0001
Heptadecanoic acid	0.19 ± 0.03	n	
9-octadecenoic acid	39.0 ± 2.69	0.23 ± 0.14	W = 744, P < 0.0001
Octadecanoic acid	5.24 ± 0.50	n	
**Wax esters**			
Octanoic acid, ethyl ester	n	y	
Nonanoic acid, ethyl ester	n	y	
Decanoic acid, ethyl ester	n	0.09 ± 0.03	
Dodecanoic acid, ethyl ester	0.005 ± 0.004	0.46 ± 0.16	W = 218, P = 0.0005
Tridecanoic acid, ethyl ester	n	0.001 ± 0.001	
Tetradecanoic acid, ethyl ester	0.01 ± 0.01	0.27 ± 0.08	W = 226, P = 0.005
Pentadecanoic acid, ethyl ester	0.01 ± 0.01	0.13 ± 0.03	W = 244, P = 0.008
Hexadecanoic acid, methyl ester	0.03 ± 0.01	0.23 ± 0.23	W = 432, P = 0.133
9-Hexadecenoic acid, ethyl ester	0.15 ± 0.10	0.31 ± 0.19	W = 274, P = 0.02
Hexadecanoic acid ethyl ester	0.55 ± 0.17	2.01 ± 0.53	W = 263, P = 0.061
Heptadecanoic acid, ethyl ester	0.003 ± 0.002	0.11 ± 0.05	W = 264, P = 0.007
Linoleic acid ethyl ester	1.11 ± 0.54	3.37 ± 1.04	W = 224, P = 0.007
9-Octadecenoic acid, ethy ester	1.29 ± 0.72	4.79 ± 1.34	W = 181, P = 0.0003
Octadecanoic acid, ethyl ester	0.23 ± 0.16	0.75 ± 0.21	W = 231, P = 0.008
**Alcohols**			
1-Pentadecanol	n	0.12 ± 0.03	
1-Hexadecanol	n	0.57 ± 0.13	
9-Octadecen-1-ol	n	0.27 ± 0.11	
1-Octadecanol	n	0.17 ± 0.06	
**Alcenes**			
1-dodecene	y	y	
**Alcanes**			
Decanal	n	y	
Tetradecanal	0.22 ± 0.04	0.21 ± 0.03	W = 327, P = 0.45
Pentadecanal	0.07 ± 0.01	0.25 ± 0.03	W = 88, p < 0.0001
Hexadecanal	0.30 ± 0.06	0.20 ± 0.04	W = 448, P = 0.20
Heptadecanal	0.06 ± 0.03	0.16 ± 0.02	W = 83, P < 0.0001
9-Octadecenal	0.01 ± 0.01	4.41 ± 1.08	W = 85, P < 0.0001
Octdecanal	0.24 ± 0.05	0.23 ± 0.02	W = 304, P = 0.25
**Sterols**			
Cholesta-3,5-diene	2.59 ± 0.44	1.34 ± 0.18	W = 467, P = 0.11
Cholesterol	1.31 ± 0.42	30.79 ± 5.86	W = 0, P < 0.0001
Cholestan-3-one	1.41 ± 0.51	15.8 ± 4.35	W = 105, P = 0.001
Cholest-4-en-3-one	0.16 ± 0.04	0.25 ± 0.06	W = 357, P = 0.80
**Others**			
Vitamin E	1.86 ± 0.31	0.35 ± 0.21	W = 679, P < 0.0001
Squalene	1.39 ± 0.35	0.16 ± 0.05	W = 654, P < 0.0001

*P values are not corrected for multiple testing^73^. Differences in abundance between pure and mixed secretions were tested using Mann-Whitney tests. Y: denotes a compound that was detected, but that was not included in the comparison between pure and mixed secretions (these compounds, being at the edge of the chromatograms, were only detected in the pure secretion samples run on the GCMS column used to analyse mixed secretion samples). n: denotes a compound that was not detected in a given type of sample. All compounds listed above had an assignment probability >88%.

When considering only LMW compounds, which are most likely to derive from bacterial fermentation (i.e., those with a molecular weight less than that of nonadecane, molecular weight: 268.5 g.mol^−1^), we detected, among samples, more compounds in the mixtures than in the pure secretions (74 vs. 59 compounds, respectively). Within individual samples, richness in LMW compounds was also greater in mixtures than in pure secretions (29.0 ± 1.compounds vs. 19.9 ± 0.8 compounds; t_1,48_ = 7.1, P < 0.0001). Moreover, for mixtures, these LMW compounds represented a significantly greater proportion of the overall chromatogram than they did for pure secretions (8.0 ± 0.8% vs. 5.8 ± 1.1%; W_31,24_ = 531, P = 0.006).

Lastly, all carboxylic acids >C_12_ occurred in greater proportion in pure secretions than in mixtures (Table 1). In contrast, all ethyl esters were in greater proportion in mixtures than in pure secretions (except hexadecanoic acid ethyl ester, W = 263, P = 0.06; Table 1). Alcohols were detected only in the mixtures (Table 1).

### Relationship between volatile compounds detected in anal-pouch mixtures and meerkat sex, social status and group membership

**Figure 2:**
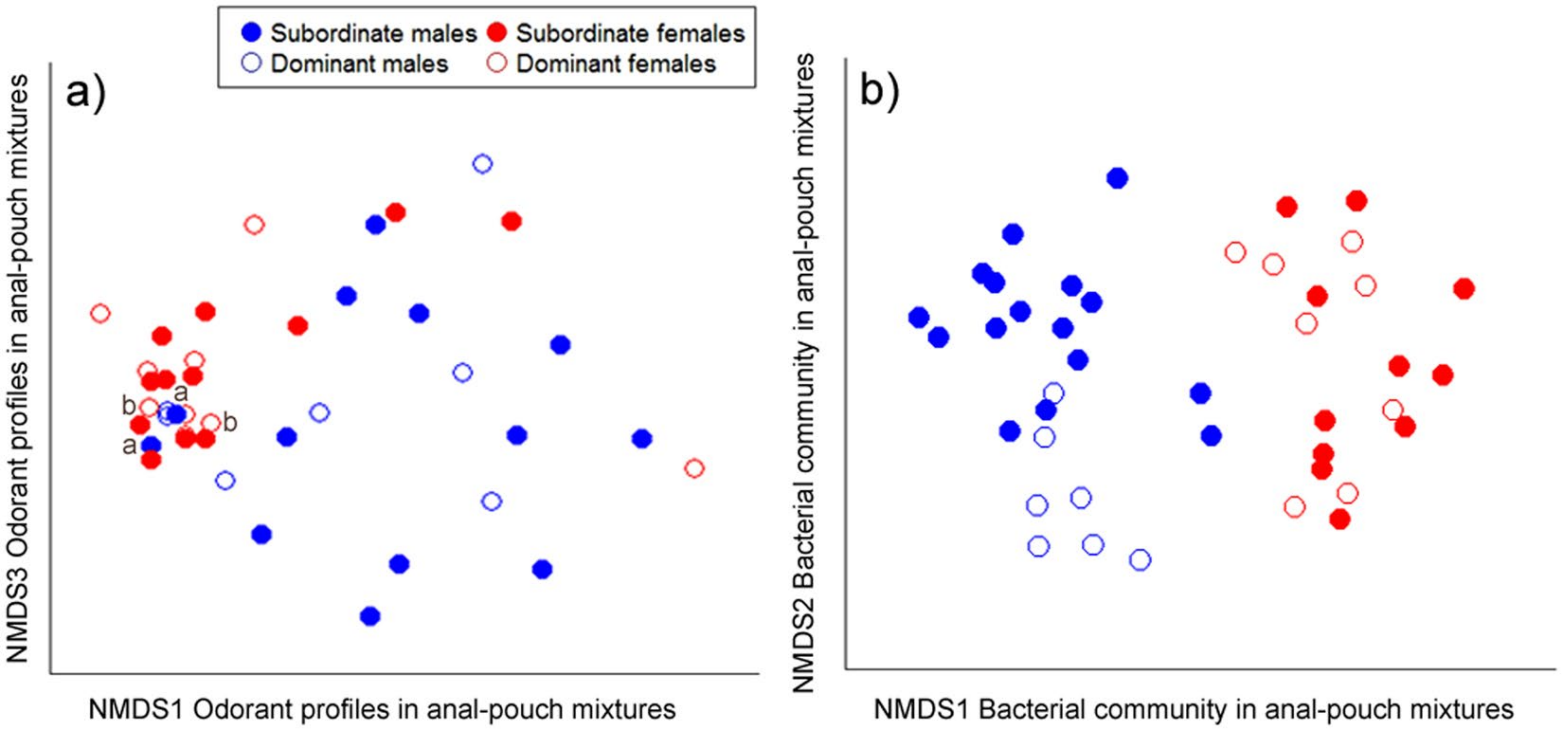
NMDS plots based on Bray-Curtis distances in (**a**) the chemical composition and (**b**) the bacterial community of anal-pouch mixtures, showing separation between meerkats by their sex and social class (2D stress of 0.12 and 0.18, for chemical composition and bacterial community, respectively). In (**a**), the two meerkats that were sampled twice are denoted by the letters a and b, referring to the nearest two solid blue and two open red symbols, respectively.

The chemical composition of LMW compounds in the 39 samples of anal-pouch mixtures obtained from dominant and subordinate meerkats varied with host sex (F_1,27_ = 5.57, P = 0.001; Fig. 2a), although not with social status (F_1,26_ = 0.74, P = 0.58; Fig. 2a) nor with the interaction between sex and social status (F_1,25_ = 0.83, P = 0.50; Fig. 2a). Using a SIMPER analysis, we showed that six compounds, including 1-hexadecanol, an unknown alcane, dodecanoic acid ethyl ester, 1-dodecene, dodecanoic acid and tetradecanoic acid (SIMPER contribution to overall dissimilarity: >4% for each of these 5 compounds), contributed most to the sex difference. Of these compounds, dodecanoic acid and tetradecanoic acid were, on average, more abundant in males than females (W = 95, P = 0.006 and W = 104, P = 0.003 respectively; relative abundances in males vs. females: 3.6 ± 0.9% vs. 0.7 ± 0.7% and 3.2 ± 0.8% vs. 0.8 ± 0.4%, respectively). By contrast, the unknown alcane and 1-dodecene were, on average, more abundant in females than males (Ps < 0.05; relative abundances in females vs. males: 6.6 ± 1.3% vs. 2.4 ± 0.7% and 50.5 ± 3.5% vs. 39.2 ± 3.5%, respectively). The relative abundance of dodecanoic acid ethyl ester and 1-hexadecanol did not differ between males and females (W = 144, P = 0.20 and W = 132, P = 0.07; relative abundances in males vs. females: 3.6 ± 1.0% vs. 2.9 ± 1.4% and 6.5 ± 1.6% vs. 1.7 ± 0.5%, respectively). We did not detect any relationship between compound richness and sex, social status or the interaction between sex and social status (data not shown).

**Figure 3:**
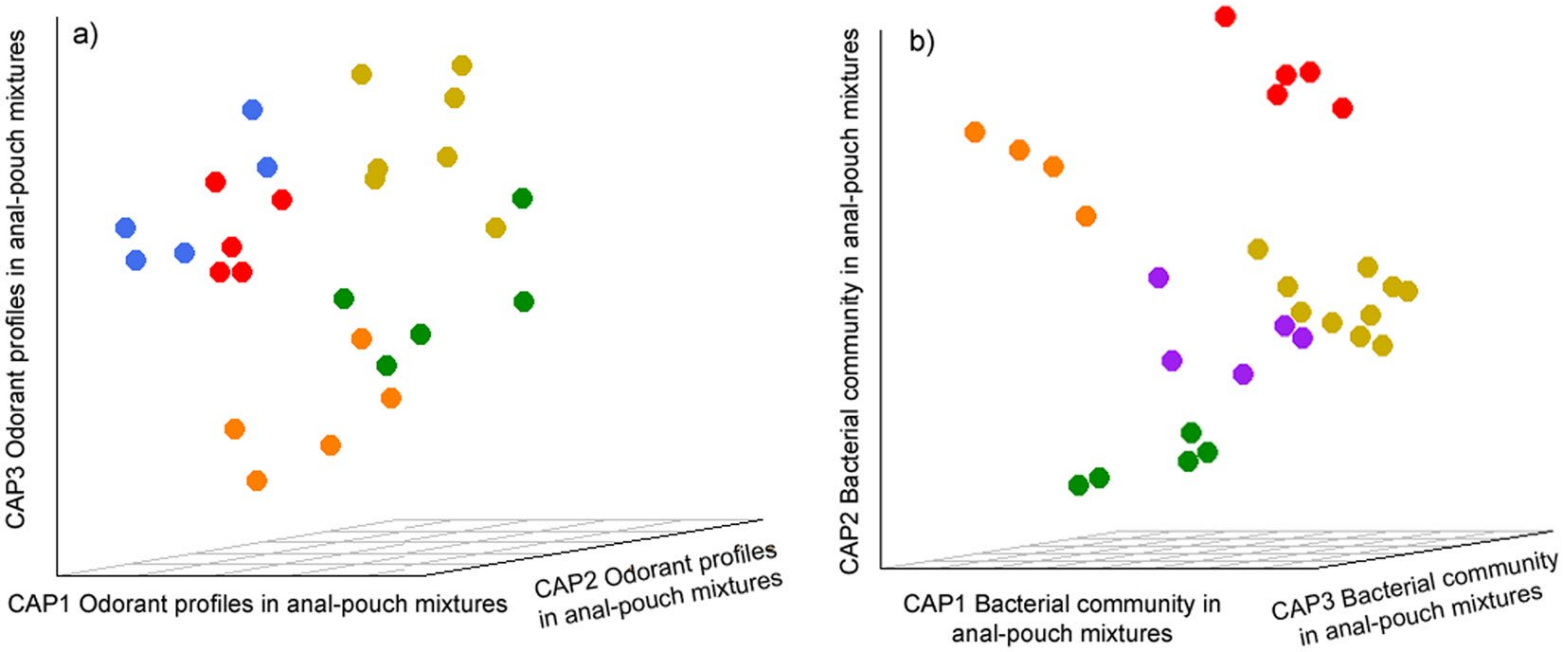
Partial distance-based redundancy analysis plots (capscale function in R) showing separation between meerkats by their group membership (plots are restricted to groups from which at least four members were sampled and for which sex and social class were partialled-out to better illustrate the separation). (**a**) Partial dbRDA based on Jaccard distance in chemical composition in anal-pouch mixtures and (**b**) partial db-RDA based on Bray-Curtis distances in bacterial communities in anal-pouch mixtures. Each colour represents a different social group.

In addition to the sex differences described above, we found evidence of group scent ‘signatures’: individuals from the same group were more similar in the chemical composition of their anal-pouch mixtures than were individuals from different groups (Jaccard distances: F_10,27_ = 1.32, P = 0.040, Fig. 3a; Bray-Curtis distances: F_10,27_ = 1.40, P = 0.068).

### Identification of bacterial assemblages in meerkat anal-pouch mixtures

**Figure 4:**
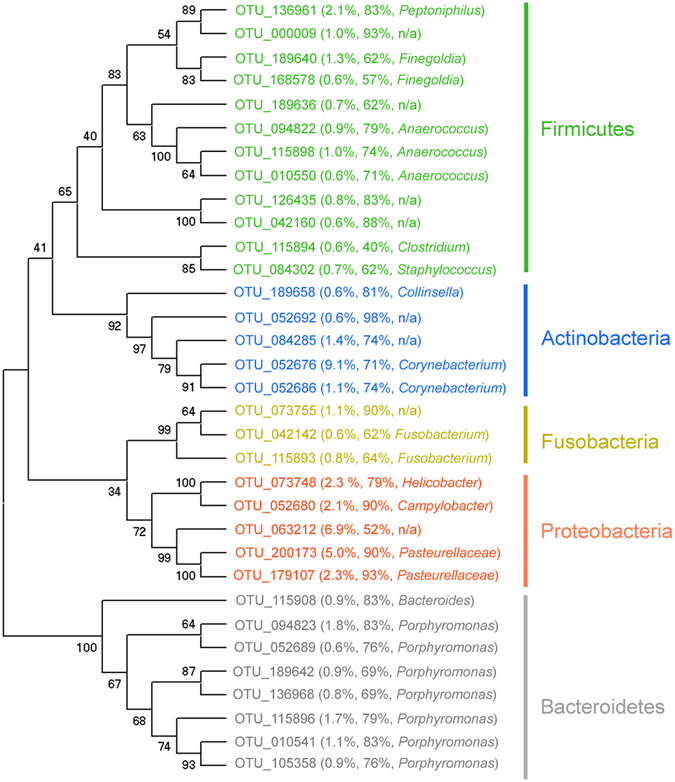
Neighbour-joining bootstrap consensus tree of the most common operational taxonomic unit (OTU) of bacteria associated with meerkat anal-pouch mixtures. The average percent abundance of all OTUs listed was >0.5%. For each of the OTUs, the respective information in parentheses indicates the average percent abundance of the OTU among the paste samples, the percentage of samples in which the OTU was found and, if applicable, the genus to which the OTU was assigned. The numbers represent the percentage of 1000 replicates (bootstrap support) for which the same branching patterns were obtained.

**Figure 5:**
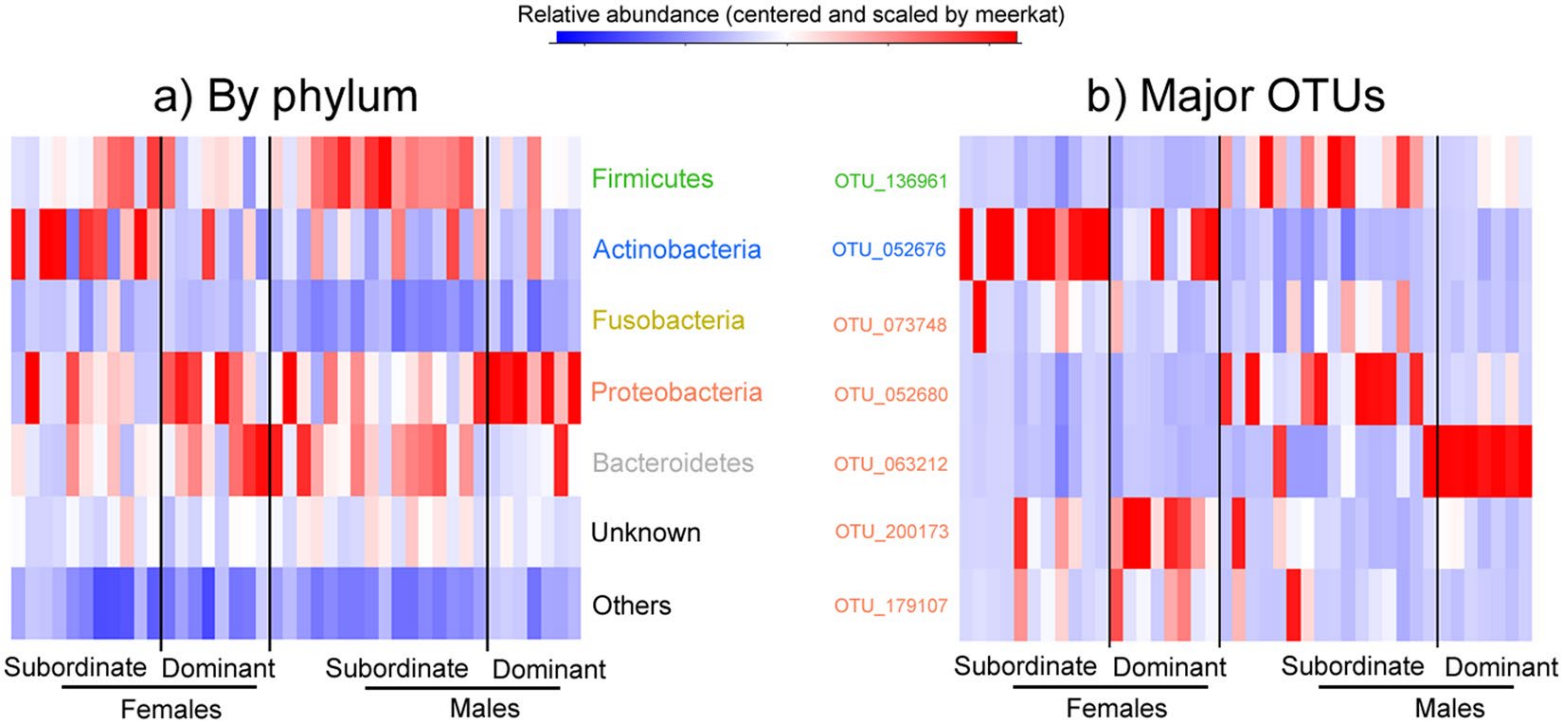
Heatmaps of the relative abundance of OTU sequences for (**a**) the different bacteria phyla and (**b**) the major OTUs (average percent abundance >2%) present in the anal-pouch mixtures of dominant and subordinate, female and male meerkats.

Considering OTUs represented by at least 10 sequences, we detected a total of 1635 OTUs in the 42 host samples obtained (Supplementary Table S1). Of these, 486 (30%) OTUs were not assigned to any phylum; the cumulative abundance of unassigned OTUs represented, on average, 11.8 ± 0.7% of the total detected. The five main bacteria phyla we detected in meerkat anal-pouch mixtures included Proteobacteria, Firmicutes, Bacteroidetes, Actinobacteria, and Fusobacteria (Figs 4 and 5; relative abundance by phylum: 27.2 ± 3.2%, 20.2 ± 1.8%, 19.0 ± 1.8%, 16.8 ± 2.8% and 3.8 ± 0.7%, respectively). The other phyla we detected represented, in total, 1.2 ± 0.3% of the relative abundance and included the following: Tenericutes (0.63%), Spirochetes (0.31%), Deferribacter (0.11%), Chlorobi (0.05%), and Acidobacteria (0.02%). We identified the seven most prominent OTUs (OTU_136961, OTU_052676, OTU_073748, OTU_052680, OTU_063212, OTU_200173 and OTU_179107), defined as having a mean relative abundance of ≥2% and as being present in ≥52% of the samples (Fig. 4). Five of these prominent OTUs were assigned to the Proteobacteria phylum, whereas one each was assigned to the Firmicutes and Actinobacteria phyla (Fig. 3).

### Relationships between bacterial assemblages and host traits

We noted several relationships between the meerkat hosts and their glandular bacterial assemblages. For instance, with regard to OTU richness, we found that female meerkats had fewer OTUs in their anal-pouch mixtures than did males (381 ± 16 vs. 445 ± 19 OTUs, F_1,40_ = 6.01, P = 0.019). By contrast, OTU richness did not vary with host social status (F_1,39_ = 1.47, P = 0.23) or group membership (F_10,28_ = 0.97, P = 0.49).

OTU composition, however, varied with all host factors studied, including with the interaction between host sex and social status (F_1,28_ = 2.45, P = 0.005; Figs 2b and 5). Notably, we observed a marked difference in OTU composition between the sexes in both subordinate and dominant meerkats (F_1,25_ = 8.39, P < 0.001 and F_1,13_ = 5.22, P < 0.001; Figs 2b and 5). Using a SIMPER analyses, we found that OTU_052676 (*Corynebacterium*), OTU_063212 (unassigned), and OTU_200173 (*Pasteurellaceae*) contributed the most to the difference between the sexes (SIMPER contribution to dissimilarity: 11%, 7%, and 5% respectively; Fig. 5b). Whereas some OTUs were more abundant in females than males (OTU_052676 in females vs. males: 20.0 ± 5.0% vs. 0.1 ± 0.0%, W = 436, P < 0.0001; OTU_200173 in females vs. males: 7.6 ± 2.3% vs. 2.9 ± 1.5%, W = 313, P = 0.02; Fig. 5b), others were more abundant in males than females (OTU_063212 in males vs. females: 12.5 ± 4.2% vs. 0.1 ± 0.1%; W = 135, P = 0.03; Fig. 5b).

Within male meerkats, subordinate and dominant individuals differed in their bacterial communities (F_1,22_ = 4.51, P < 0.001; Figs 2b and 5) and, based on SIMPER analyses, OTU_063212 contributed the most to this status difference (SIMPER contribution: 20%; average abundance ± SE: 34.3 ± 8.7% in dominant males vs. 3.0 ± 2.1% in subordinate males; W = 7, P < 0.001; Fig. 5b). In contrast, we did not detect differences in the bacterial communities of dominant and subordinate females (F_1,17_ = 1.49, P = 0.068, Fig. 2b).

**Figure 6:**
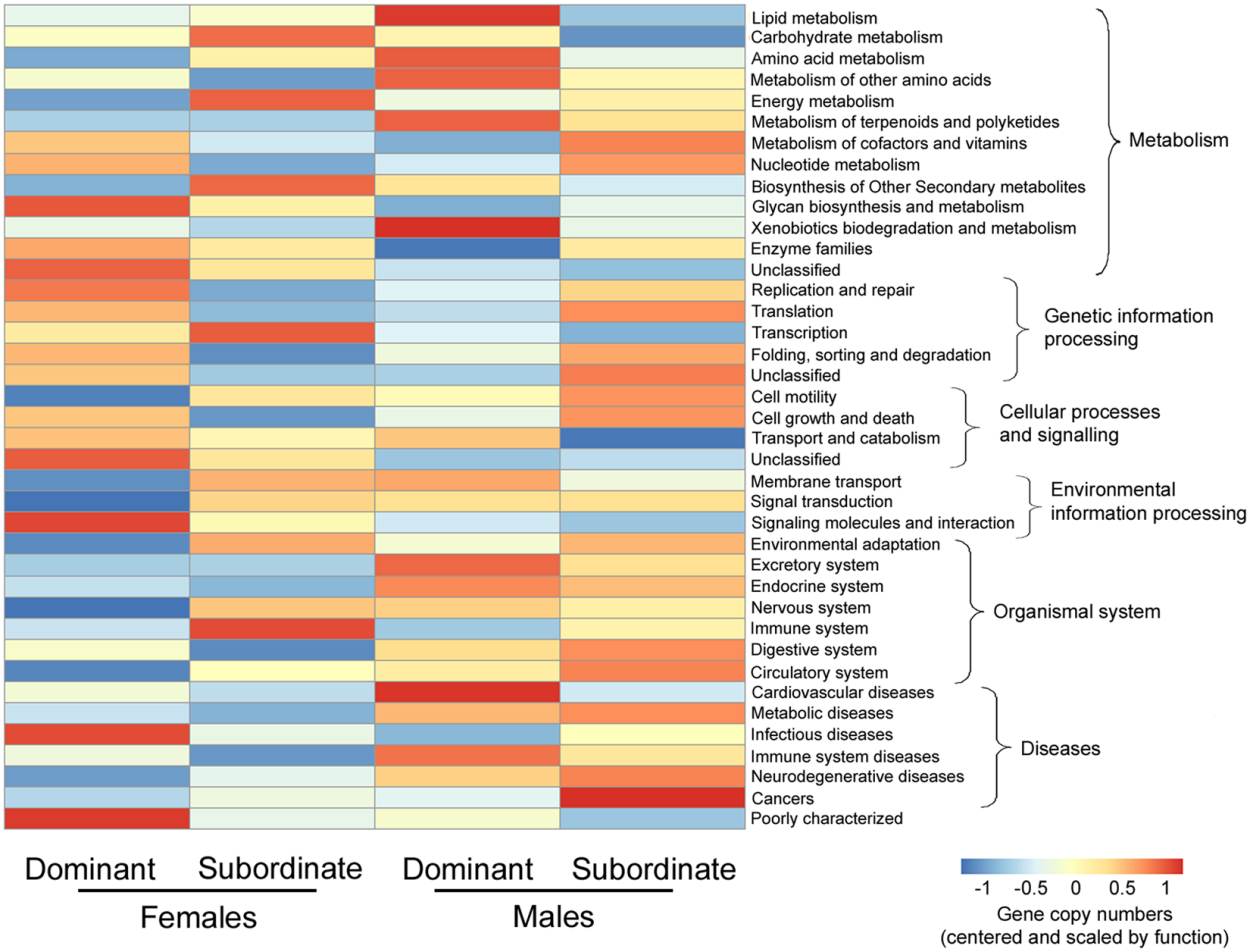
Predicted functions of microbiota in meerkat paste and how they might differ between dominant and subordinate males and females. KEGG pathway of the second levels is shown in the heatmap.

By examining the two-level Kyoto Encyclopedia of Genes and Genomes (KEGG) pathways of bacteria in meerkat anal-pouch mixtures (Fig. 6), we found that genes controlling lipid metabolism were enriched in dominant males compared to subordinate males and to females of either social class (sex*social status: F_1,38_ = 11.0, P = 0.003; all Tukey post hoc tests comparing dominant males with other meerkat categories: P < 0.04).

Lastly, consistent with prior findings based on DNA fingerprint analyses of similar samples^23^, the composition of bacterial communities in anal-pouch mixtures also varied with the host’s group membership (F_10,28_ = 1.28, P = 0.022; Fig. 3b).

### Covariance between volatile chemicals and bacterial communities in meerkat anal-pouch mixtures

As anticipated for the anal-pouch mixtures of meerkats, chemical composition covaried with bacterial composition (Bray-Curtis distances: r = 0.17, Mantel test: P = 0. 004, Jaccard distances: r = 0.09, P = 0.10; n = 30 samples). To identify the bacteria that drove this correlation, we performed a “bv.step” analysis and found that eight OTUs best explained the chemical composition of anal-pouch mixtures. These OTUs belonged to the genus *Porphyromonas* (OTU_094823, OTU_105358, OTU_000025, OTU_000038), *Anaerococcus* (OTU_158062), *Fusobacterium* (OTU_126442), and *Campylobacter* (OTU_052680).

**Figure 7:**
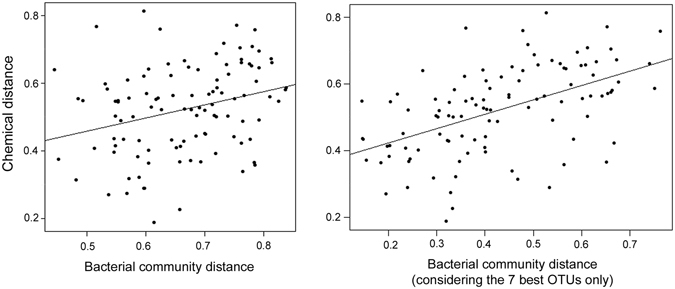
Chemical distance in relation to bacterial distance in male meerkat anal paste mixtures. Bacterial distance was calculated using (**a**) all OTUs or (**b**) the 7 OTUs that best explained the males’ chemical profiles.

To test for any host sex differences in covariation between the volatile chemicals and bacterial assemblages, we performed separate Mantel tests for males and females. Chemical and bacterial composition covaried positively in males (Bray-Curtis distances: r = 0.29, P = 0.008, Jaccard distances: r = 0.15, P = 0.13; n = 15 males, Fig. 7a), but not in females (r = 0.07, P = 0.28, n = 15 females). Using a “bv. Step” analysis in males, we showed that a subset of seven OTUs best explained the chemical composition of their anal-pouch mixtures (Rho = 0.67; Fig. 7b). These seven OTUs were either unclassified (OTU_031640 and OTU_052716) or belonged to the genus *Porphyromonas* (OTU_189642, OTU_136968, and OTU_000025), *Anaeroccocus* (OTU_158062), and *Firmicutes* (OTU_136961).

## Discussion

Coupling deep sequencing of the bacterial 16 S rRNA gene with chemical analyses of meerkat glandular secretions, we provide a rare exploration of the patterns within and relationships between the host’s volatile chemical signals and its bacterial assemblages. Meerkat anal paste, whether deriving from the gland directly (i.e., in purer form) or from the anal pouch (i.e., as more of a mixture), was rich in volatile chemicals, including derivatives that likely owed to bacterial metabolism. Nevertheless, consistent with expectations of increased fermentation occurring in the anal pouch, relative to within the anal gland, we detected more LMW volatiles associated with paste obtained from the pouch than from the gland. Most notably, volatile chemicals contained within anal-pouch mixtures covaried with the host’s bacterial assemblages; moreover, both the chemical and bacterial profiles of meerkats varied by host sex and group membership. These results are consistent with a role for fermentative bacteria in the production of meerkat scents – scents that encode information that is socially relevant to the host.

The greater occurrence of LMW volatiles in anal-pouch mixtures than in pure glandular secretions may reflect the breakdown of long-chain compounds into smaller volatiles. Such metabolism could involve various biochemical processes, including the actions of integument enzymes, oxidation^25^ and microbial activity. In particular, compared to pure glandular secretions, anal-pouch mixtures contained several alcohols, as well as reduced proportions of fatty acids (>C_12_), but they had increased proportions of the corresponding wax esters. Patterns of differential chemical composition between the two forms of paste may owe to the esterification of glandular fatty acids after paste is secreted into the pouch. Interestingly, in mice and humans, wax monoester biosynthesis has been suggested to involve fatty acids as starting materials, followed by reduction to linear alcohols, with final conversion of the fatty alcohols to wax monoesters by wax synthase enzymes^26^. Despite that wax synthases can originate from the host^27–29^, they are also widespread in bacteria^30–34^. Given that esters are generally more volatile than are carboxylic acids of similar weight, bacterial esterification of endogenous fatty acids might provide the host with a mechanism to increase the volatility of its glandular secretions, potentially improving its odour perception by conspecifics.

Identification of the bacterial phylotypes via deep sequencing allowed us to show that the most abundant bacteria phyla in meerkat anal-pouch mixtures are similar to those detected in the glandular secretions of other scent-marking mammals^35–37^. Meerkat anal-pouch mixtures harboured several corynebacteria, anaerococci, and *Porphyromonas* whose relatives are known to produce odorants, some of which are functional semiochemicals. For example, some corynebacteria have a lipid-catabolizing function and can produce volatile fatty acids from methyl-branched fatty acids^31^. Others can also form long-chain esters from alcanes^38^. Researchers performing both correlative and experimental studies have shown that corynebacteria and Anaerococci can cleave odorant precursors present in the human armpit and lead to the release of short-branched fatty acids that are key components of axillary odour^39, 40^. Some *Porphyromonas*, such as the periodontal and endodontal human pathogens *Porphyromonas gingivalis* and *Porphyromonas endodontalis*, are known to produce carboxylic acids and methylated carboxylic acids^41, 42^. Lastly, bacteria from the phyla Proteobacteria and Firmicutes can produce C_9_–C_18_ carboxylic acids and some of the corresponding methyl esters that are potent odorous stimulants for oviposition in gravid mosquitoes^43^.

Most importantly, both the odorants and the bacterial communities in meerkat anal-pouch mixtures varied with host sex and group membership. With regard to the bacterial patterns, these findings replicate and extend previous results obtained using a DNA fingerprint method^23^. Sex-specific odorants or odour mosaics are widespread across animal taxa^10, 44–47^ and have been detected in several species of mongooses^9, 48^. In meerkats, these sex differences in signal production or expression are congruent with the meerkats’ differential behavioural responses to the presentation of anal-pouch mixtures from conspecific females and males (Leclaire, unpublished data). Some of the major compounds contributing to the sex differences in anal-pouch odorants include 1-hexadecanol, dodecanoic acid and dodecanoic acid ethyl ester. The greater abundance of these compounds in anal-pouch mixtures than in pure glandular secretions might also reflect a bacterial origin, consistent with the meerkats’ sex difference in bacterial assemblages.

These sex differences in the hosts’ microbiota owed mainly to a *Corynebacterium* (OTU_052676) being in greater proportion in females than males, and to a proteobacterium (OTU_063212) being in greater proportion in males than females. Beyond these sex differences, covariation across sexes between the chemical composition and microbiota of anal-pouch mixtures suggests a bacterial mechanism to sex-specific odorants in meerkats. Across sexes, four of the eight OTUs that best explained the chemical composition of anal-pouch mixtures belonged to the genus *Porphyromonas*, some of whose members are known to generate volatile fatty acids^41^ or oral malodour in humans^49^.

As detected in wild hyaenas^17^, we also found that the bacterial assemblages and volatile chemical profiles covaried among individual meerkats, albeit principally within males. At an ultimate level, benefits of harbouring odour-producing bacteria may differ between males and females. In meerkats, for instance, the benefits of deterring intruders from foreign groups are highly biased towards males^50^, suggesting that olfactory advertisement of group membership may be more important for males than females^18, 51^. Male meerkats also disperse in adulthood and may change group affiliation several times over the course of their lifespan. Therefore, males may require greater flexibility than females in their olfactory advertisement of group membership. At a proximate level, such olfactory flexibility may be achieved via a bacterial mechanism of scent production, as has been suggested to explain the existence and acquisition of group scent signatures in other species^35, 52, 53^. An anal-pouch microenvironment favourable to odour-producing bacteria thus may be under stronger selection in male meerkats than in females. In support of this interpretation, we found that bacterial genes controlling lipid metabolism were enriched in dominant males compared to females and subordinate males. These genes can code for enzymes that synthesize lipids incorporated in bacterial membranes^54^ or that are used for defense or cell-to-cell communication^55, 56^. Greater abundance of genes involved in lipid metabolism in dominant males might therefore correlate with increased bacterial production of volatile lipids and derivatives. The breakdown of long-chain compounds into smaller volatiles was studied in subordinate meerkats only, so we cannot exclude the possibility of more intense degradation of long-chained compounds in dominant male meerkats.

Consistent with the fermentation hypothesis for chemical signalling, we have emphasized the potential role of bacteria in producing meerkat olfactory cues; however, the correlative nature of the evidence does not preclude alternative interpretations. For instance, individual or group differences may originate in the chemical composition of the anal secretions, produced endogenously, which could then lead to differences in the microbiota that can flourish and be detected. Bacterial establishment and growth depend on the physical and chemical parameters of the microenvironments they encounter, including the organic compounds found at the surface of the host’s body. For instance, several fatty acids, such as oleic acid, which is detected in meerkat anal paste, can either promote or inhibit the growth of various pathogenic and commensal bacteria^57, 58^. Alternately, host production of chemical compounds might be determined by exposure to specific bacteria^59^. In great tits (*Parus major*), the size of the scent gland and the composition in glandular wax esters vary with experimentally increased bacterial load on the feathers^59^, suggesting that investment in scent compounds may be adjusted to bacterial load or bacterial community assemblage.

Lastly, although bacteria are known to emit LMW organic volatiles (<120D)^24^, our chemical methods did not allow us to detect volatiles smaller than octanoic acid (molecular mass: 144 D). Therefore, future studies focusing on LMW volatiles in meerkat anal-pouch mixtures, combined with *in-vitro* cultures of the specific bacterial strains found in the mixtures and *in-vivo* experimental manipulations of bacterial communities, will be required to determine to what extent bacteria produce the compounds used by meerkats to communicate with their conspecifics.

## Materials and Methods

### Study site and subjects

This study was conducted on the adult members of a wild population of meerkats in the Kuruman River Reserve (KRR; 26°58′S, 21°49′E), which is situated on ranch land, composed of vegetated sand dunes, in the southern Kalahari of South Africa. Details about this site have been published previously^60^.

The meerkats at this site are habituated to close observation by humans. Individuals are implanted with subcutaneous transponder chips and are recognizable in the field by unique dye marks applied by hand to the fur of awake animals^18^. At least one animal per group is fitted with a radio collar (Sirtrack Havelock North, New Zealand) to facilitate locating groups. Each group is visited approximately once every three days to record all key life-history events, including group movements and changes in group composition or individual dominance status.

Our focal animals for chemical analyses (n = 37 individuals; Supplementary Table S1) included 12 subordinate females (age: 689 ± 79 days, range: 366–1405 days), 8 dominant females (age: 2312 ± 206 days, range: 1111–2929 days), 12 subordinate males (age: 776 ± 97 days, range: 312–1190 days) and 7 dominant males (age: 1606 ± 219 days, range: 1160–2524 days). Of our focal animals, most (n = 30; Supplementary Table S1) also served as subjects for bacterial analyses, and included 8 subordinate females, 7 dominant females, 8 subordinate males and 7 dominant males. All of the protocols were approved by Duke University’s Institutional Animal Care and Use Committee (protocol registry numbers: A171-09-06 and A143-12-05) and by the University of Pretoria’s Animal Use and Care Committee (ethical approval number: EC074-11, to C.M.D.). Our methods were carried out in accordance with the approved guidelines.

### Sample collection

As in Leclaire *et al*.^23^, we collected two versions of anal paste for chemical analyses: (1) From March 2011 to November 2011, we collected ‘pure’ anal gland secretions when meerkats were captured and anaesthetized during the course of other studies. We partially everted the anal pouch, gently pressed the anal gland and collected the exudate in 2-ml PTFE-faced septum glass vials. We collected 10 samples from subordinate females and 21 samples from subordinate males (Supplementary Table S1). (2) In November 2011, we also collected ‘mixed’ anal-pouch secretions by rubbing precleaned cotton swabs against the interior wall of the anal pouch of awake, freely behaving, meerkats that were resting near their burrow entrance. We sampled most (n = 37; Supplementary Table S1) individuals only once, but sampled an additional two individuals twice. We set aside one blank cotton swab, in the field, to serve as a control in the chemical analyses (see below). All odorant samples were transferred from the KRR field site to the laboratory in a cool box filled with ice packs. They arrived refrigerated and were then kept frozen at −20 °C until analysis.

Also as in Leclaire *et al*.^23^, we collected only the mixed form of anal paste for bacterial analyses: we rubbed sterile cotton swabs (Copan sterile plain swabs; Copan Italia, Brescia, Italy) against the interior wall of the anal pouch of awake, freely behaving, meerkats that were resting near their burrow entrance. Swab samples (n = 44; Supplementary Table S1) were stored at −20 °C until analyses. On average, we obtained the samples destined for chemical and bacterial analyses, respectively (see below), from a given individual within an interval of 10 ± 2 days (range: 0–26 days).

### Chemical analyses

We extracted the volatile organic compounds from both pure and mixed paste following a protocol adapted from Safi and Kerth^53^. We added 500 µl of deionized water and 500 µl of methyl-tert-butyl ether (MTBE) to each sample, vortexed the vials for 45 s, and then transferred the cotton swab and solvent into a 4-ml glass vial. We then centrifuged the vials for 5 min at 3000 rpm. We removed the solvent fraction with a pipette and placed it into a clean, solvent-washed chromatography vial. This extraction procedure was repeated twice, each time with 500 µl of MTBE added to the cotton swab. We then placed a 1-ml aliquot of the solvent fraction into a clean, conical vial and concentrated those samples over compressed nitrogen to a final volume of 50–100 µl. We kept all the samples on ice throughout the entire procedure to minimise the loss of LMW volatiles. We added 5 µl of hexachlorobenzene to each vial to serve as an internal standard.

We analysed these samples via gas chromatography and mass spectrometry (GCMS), using a Shimadzu GCMS-QP2010 (Shimadzu Scientific Instruments, Columbia, MD) equipped with an AOC-20 series autosampler and a Restek SHR5XLB (30 m × 0.25 mm × 0.25 µm, Shimadzu) capillary column. Helium was used as the carrier gas, at a constant linear velocity of 1 ml/min. The injector was set at 280 °C and the ion source was held at 200 °C. Masses were scanned from 50–525 *m/z* in electron ionization mode. We injected 1 µl of the odorant solution in splitless mode. We ran the following temperature protocol after a 3-min solvent delay: 80 °C–180 °C ramped at 20 °C/min; 180 °C–320 °C ramped at 5 °C/min (held at 320 °C for 7 min).

We ran the samples in large batches to minimise potential interassay variability and regularly interspersed blanks throughout the sample analyses. Because we ran the two types of odorant samples on separate, but identical GCMS columns, we ran one sample twice, using each of the columns, to determine the range of overlap in the compounds detected on both columns. We then used that range for our statistical analyses (see below) of the compounds detected (Table 1). Identification of compounds was based on mass spectral fragmentation pattern, using the chromatographic retention index and the NIST and Wiley mass spectral libraries.

### Bacterial analyses

We extracted DNA only from the mixed samples (derived from meerkat anal pouches) using a WIZARD Genomic DNA Purification Kit (Promega, Lyon, France), following the protocol described in Leclaire *et al*.^23^. Two control samples, that had been collected in the field by opening the swab tubes for a few seconds, were extracted using the same protocol.

PCR amplifications were performed in 30-μL mixtures containing 3 μL of diluted DNA extract. Each PCR mixture was composed of 1U of AmpliTaq Gold DNA Polymerase (Applied Biosystems, Foster City, CA, USA), 2.5 mM of MgCl_2_, 1x of Taq Buffer, 0.2 mM of each dNTP and 2.4 ng of bovine serum albumin (Promega Corporation, Madison, USA). PCR conditions consisted of an initial denaturation at 95 °C for 5 min, followed by 35 cycles of denaturation (at 95 °C for 30 s), annealing (at 57 °C for 30 s) and elongation (at 72 °C for 30 s). We used universal primers that specifically amplified the v5-6 region (ca 295 bp length) of the bacterial 16 S rRNA gene (BACTB-F: GGATTAGATACCCTGGTAGT; and BACTB-R: CACGACACGAGCTGACG)^61^. To discriminate samples after sequencing, we labelled both forward and reverse primers at the 5′ end with a combination of two different 8 bp tags. The PCR products were purified, using the QIAquick PCR purification Kit (Qiagen GmbH, Hilden, Germany), and then pooled. Amplicons were then sequenced with an Illumina MiSeq platform, using the 2 × 250 bp protocol (Fasteris SA, Plan-les-Ouates, Switzerland). We included PCR blank controls in the sequenced multiplex to detect potential reagent contaminants.

We analysed the sequence reads following Taberlet and colleagues^62^, with some adjustments, using OBITools package^63^. Briefly, after assembly of paired-end reads, we assigned reads to their respective samples (with 0 and 2 mismatches allowed on tag and primer sites, respectively) and excluded reads with low assembly scores or that contained ambiguous bases (i.e., “N”). We dereplicated strictly identical reads using the “obiuniq” algorithm and removed singletons (i.e., one single occurrence over the entire dataset, which likely indicates degraded sequences). We used the “obiclean” algorithm to detect and remove potential PCR/sequencing errors (Boyer *et al*. 2015). We then clustered the remaining sequences into Operational Taxonomic Units (OTUs) based on their similarity using the “sumaclust” algorithm (https://git.metabarcoding.org/obitools/sumatra/wikis/home), with a 97% similarity threshold. Taxonomic assignations were obtained using “ecotag” with the MOTHUR version of the SILVA bacterial database (release 102). We considered only the taxonomic assignation of the most abundant sequence of each OTU. Lastly, low abundance false positives have been repeatedly observed as a consequence of “tag-switching” events^64, 65^. We therefore removed all OTUs that had a total read abundance of <10 reads. We used PICRUSt (Phylogenetic Investigation of Communities by Reconstruction of Unobserved States) to predict metagenome functional content from 16S rRNA^66^.

OTU count data were standardized through conversion into intra-sample relative abundances, and the contribution of highly prominent OTUs to quantitative similarity index calculation was tempered by square-root transformations of the data. One subordinate male and one subordinate female had few bacterial sequences (2 and 12 sequences, respectively) in their samples, compared to the other individuals (mean ± SE: 4942 ± 192 sequences; range: 2104–8117 sequences), and were therefore excluded from the analyses. We excluded 241 OTUs from the analyses because they were more abundant in the PCR-specific controls than in the samples, and we excluded an additional 15 OTUs because they were more abundant in the sampling-specific controls than in the samples. The number of sequences did not differ between males and females (4942 ± 288 sequences vs. 4942 ± 264 sequences respectively; F_1,40_ = 0.01, P = 0.93) and between subordinates and adults (5100 ± 187 sequences vs. 4676 ± 410 sequences; F_1,41_ = 1.14, P = 0.29). OTUs sequences have been deposited in GenBank (accession numbers: KY630752–KY631487; Supplementary Table S2).

### Data handling and statistical analyses

For statistical analyses of our chemical data, we excluded peaks that were present in only one sample. Also, because we could not control for the amount of secretion collected, regardless of sampling method, we did not rely on the absolute abundance of chromatogram peaks; rather, we quantified each peak as the proportion of the peak size relative to the total area of the chromatogram^67^. For odorant samples obtained from the anal pouch, we first verified that repeat samples from the same individual produced consistent chromatograms (see Fig. 2) and then retained only the most-recently obtained sample in our statistical analyses to avoid pseudoreplication.

We used a subset of the chemical data (derived from anal-pouch samples) in our correlative analyses against the bacterial dataset. This is because most organic compounds produced by bacteria are in the range of 100–250 D^24^ and, although some bacteria are known to metabolize cholesterol into coprostanol^68^, sterol biosynthesis is mainly viewed as an eukaryotic process^69^. We therefore restricted our analyses of chemical and bacterial covariation to those chemical compounds that had lower retention time than that of nonadecane (C_19_H_40_, molecular weight: 268 D). In addition, because pure anal-gland secretions were sampled from subordinates only, we restricted the comparison between pure secretions and mixtures to subordinate meerkats.

We performed all statistical analyses using the R statistical software^70^ and express values as mean ± standard error (SE) throughout. To determine if the chemical composition or the bacterial communities present in the meerkat’s anal pouch related to the animal’s sex and social class (i.e., subordinate versus dominant), to the two-way interaction between these two factors, or to the animal’s group membership, we used a PERMANOVA with 5000 permutations i.e. nonparametric multivariate analysis of variance, Adonis function, VEGAN package in R^71^, based on Bray-Curtis distance for relative abundance data and Jaccard distance for presence/absence data. Whenever we observed differences between the sexes or social classes in either chemical composition or bacterial community, we conducted similarity percentage analyses (SIMPER procedure in the VEGAN package in R) to elucidate the contribution of specific chemical compounds or OTUs. We used linear models to determine if OTU richness and chemical compound richness were related to the animal’s sex, social class, the two-way interaction between these two factors, or to the animal’s group membership.

We evaluated covariance between chemical compounds and bacterial assemblages in mixed paste using Mantel tests (VEGAN package in R). When the covariance was significant, we used the “bv.step” procedure in R equivalent to the BVSTEP procedure in BEST in the software PRIMER^72^ to select the combination of OTUs that best explained the chemical composition of odorants. All of the results described herein are based on Bray-Curtis distances and, unless otherwise stated, are similar to results based on Jaccard distances.

## Electronic supplementary material

Metadata Table S1

Supplementary Table S2
