# Supplementary material for: Social odours covary with bacterial community in the anal secretions of wild meerkats
Source: Sci Rep. 2017 Jun 12;7:3240. doi: 10.1038/s41598-017-03356-x (PMC5468246; doi:10.1038/s41598-017-03356-x)
Supplement: Supplementary file 1 — Metadata Table S1 [file 41598_2017_3356_MOESM1_ESM.pdf]

Social odours covary with bacterial community in the anal secretions of wild meerkats

Sarah LECLAIRE, Staffan JACOB, Lydia K. GREENE, George R. DUBAY & Christine M. DREA

Supplementary Table S1: Metadata describing the social status, sex, group membership, and samples collected for each meerkat used in the study. The label of the vial where the swab sample was saved for bacterial analyses is given (for association with Supplementary Table S2). Empty cells mean that no sample was collected. The two bacterial samples that had few OTUs and were excluded from the analyses are not shown.

Meerkat ID	Group ID	Social status	Sex	Bacterial sample	Odorant sample	
				Anal-pouch mixtures	Anal-pouch mixtures[a]	Pure glandular secretions[a]
Ind1	GroupA	A	F		x	
Ind10	GroupB	A	M	A84	x	
Ind11	GroupJ	D	F	A40	x	
Ind12	GroupD	D	F	A43	x	
Ind13	GroupD	A	F	A24	x	
Ind14	GroupD	A	F			x
Ind15	GroupD	D	M	A09	x	
Ind16	GroupD	A	M			x
Ind17	GroupD	A	M	A31		x
Ind18	GroupD	A	M	A59		x
Ind19	GroupE	A	F			x
Ind2	GroupA	A	F			x
Ind20	GroupG	D	F		x	
Ind21	GroupF	A	F		x	
Ind22	GroupF	A	F	A67	x	
Ind23	GroupF	A	F	A69	x	
Ind24	GroupF	D	M	A80	x	
Ind25	GroupK	D	M	A38	x	
Ind26	GroupF	A	M	A39		
Ind27	GroupF	A	M			x
Ind28	GroupF	A	M			x
Ind29	GroupG	A	F	A28	x	
Ind3	GroupA	A	M	A60	x	
Ind30	GroupG	A	F			x

Ind31	GroupG	A	F			x
Ind32	GroupG	A	M		x	
Ind33	GroupG	A	M			x
Ind34	GroupG	A	M			x
Ind35	GroupG	A	M		x	
Ind36	GroupF	D	F	A46	x	
Ind37	GroupH	D	F	A23	x	
Ind38	GroupH	A	F	A35	x	
Ind39	GroupH	A	F	A02		
Ind4	GroupA	D	M	A50	x	
Ind40	GroupH	A	F		x	
Ind41	GroupM	D	M	A71	x	
Ind42	GroupH	A	M		x	
Ind43	GroupC	A	M	A86		
Ind44	GroupC	A	M	A83	x	
Ind45	GroupH	A	M	A04		
Ind46	GroupH	A	M			x
Ind47	GroupI	A	F			x
Ind48	GroupJ	A	F	A36		
Ind49	GroupJ	A	M	A08	x	
Ind5	GroupA	A	M	A63	x	x
Ind50	GroupJ	A	M			x
Ind51	GroupK	A	F	A65	x	
Ind52	GroupK	D	F	A57		
Ind53	GroupL	A	F			x
Ind54	GroupL	A	F			x
Ind55	GroupL	A	F		x	x
Ind56	GroupL	A	F			x
Ind57	GroupL	A	M			x
Ind58	GroupL	A	M			x
Ind59	GroupL	A	M			x
Ind6	GroupA	A	M			x
Ind60	GroupL	A	M			x
Ind61	GroupL	A	M	A18		
Ind62	GroupL	A	M		x	
Ind63	GroupL	A	M			x
Ind64	GroupL	A	M			x
Ind65	GroupA	D	F	A19	x	
Ind66	GroupL	D	F	A03	x	
Ind67	GroupM	D	F	A11	x	
Ind68	GroupM	A	F	A72	x	
Ind69	GroupM	A	F	A27		
Ind7	GroupA	A	M			x
Ind70	GroupM	A	F	A56	x	
Ind71	GroupG	D	M	A49	x	
Ind72	GroupH	D	M	A01	x	

Ind73	GroupM	A	M	A73	x	
Ind74	GroupM	A	M	A78	x	
Ind75	GroupM	A	M	A51		x
Ind76	GroupM	A	M	A70	x	
Ind77	GroupM	A	M	A54		
Ind8	GroupB	A	M			x
Ind9	GroupB	A	M			x

[a] x: an odorant sample was collected.
